# A genomic perspective on the taxonomy of the subtribe Carcharodina (Lepidoptera: Hesperiidae: Carcharodini)

**DOI:** 10.11646/zootaxa.4748.1.10

**Published:** 2020-03-05

**Authors:** JING ZHANG, ERNST BROCKMANN, QIAN CONG, JINHUI SHEN, NICK V. GRISHIN

**Affiliations:** 1 Howard Hughes Medical Institute, University of Texas Southwestern Medical Center, 5323 Harry Hines Blvd, Dallas, TX, USA 75390-9050; 2 Departments of Biophysics and Biochemistry, University of Texas Southwestern Medical Center, 5323 Harry Hines Blvd, Dallas, TX, USA 75390-9050; 3 present address: Institute for Protein Design and Department of Biochemistry, University of Washington, 1959 NE Pacific Street, HSB J-405, Seattle, WA, USA 98195; 4 Laubacher Str. 4, 35423 Lich, Hessen, Germany

**Keywords:** taxonomy, genomic sequencing, phylogeny, grizzled skippers, marbled skippers

## Abstract

We obtained whole genome shotgun sequences and phylogenetically analyzed protein-coding regions of representative skipper butterflies from the genus *Carcharodus* Hübner, [1819] and its close relatives. Type species of all available genus-group names were sequenced. We find that species attributed to four exclusively Old World genera (*Spialia* Swinhoe, 1912, *Gomalia* Moore, 1879, *Carcharodus* Hübner, [1819] and *Muschampia* Tutt, 1906) form a monophyletic group that we call a subtribe Carcharodina Verity, 1940. In the phylogenetic trees built from various genomic regions, these species form 7 (not 4) groups that we treat as genera. We find that *Muschampia* Tutt, 1906 is not monophyletic, and the 5th group is formed by currently monotypic genus *Favria* Tutt, 1906 **new status** (type species *Hesperia cribrellum* Eversmann, 1841), which is sister to *Gomalia*. The 6th and 7th groups are composed of mostly African species presently placed in *Spialia*. These groups do not have names and are described here as *Ernsta* Grishin, **gen. n.** (type species *Pyrgus colotes* Druce, 1875) and *Agyllia* Grishin, **gen. n.** (type species *Pyrgus agylla* Trimen, 1889). Two subgroups are recognized in *Ernsta*: the nominal subgenus and a new one: *Delaga* Grishin, **subgen. n.** (type species *Pyrgus delagoae* Trimen, 1898). Next, we observe that *Carcharodus* is not monophyletic, and species formerly placed in subgenera *Reverdinus* Ragusa, 1919 and *Lavatheria* Verity, 1940 are here transferred to *Muschampia*. Furthermore, due to differences in male genitalia or DNA sequences, we **reinstate** *Gomalia albofasciata* Moore, 1879 and *Gomalia jeanneli* (Picard, 1949) as species, not subspecies or synonyms of *Gomalia elma* (Trimen, 1862), and *Spialia bifida* (Higgins, 1924) as a species, not subspecies of *Spialia zebra* (Butler, 1888). Sequencing of the type specimens reveals 2.2–3.2% difference in COI barcodes, the evidence that combined with wing pattern differences suggests a **new status** of a species for *Spialia lugens* (Staudinger, 1886) and *Spialia carnea* (Reverdin, 1927), formerly subspecies of *Spialia orbifer* (Hübner, [1823]).

## Introduction

Checkered, grizzled and marbled wing patterns are present in a number of Hesperiidae species from around the world (Evans, 1937, 1949, 1953). Previously considered close relatives (Evans, 1937, 1949), they have since been placed in three different tribes (Li et al., 2019; Warren, Ogawa, & Brower, 2008, 2009). Checkered skippers are confusingly close to each other in their wing patterns, but the reasons for such similarity are unclear. African *Alenia* Evans, 1935 belongs to the tribe Celaenorrhinini Swinhoe, 1912. Old World *Spialia* Swinhoe, 1912 and *Muschampia* Tutt, 1906 are placed in the tribe Carcharodini Verity, 1940. Holarctic *Pyrgus* Hübner, [1819] with its New World relatives *Burnsius* Grishin, 2019, *Chirgus* Grishin, 2019 and *Heliopetes* (*Heliopyrgus*) *americanus* (Blanchard, 1852) are from the tribe Pyrgini Burmeister, 1878. These 5 genera and one species are more similar to each other in appearance than to their closest relatives. Moreover, some species of *Muschampia* are more marbled than checkered and their patterns indeed remind of *Carcharodus* Hübner, [1819], the nominal genus of their tribe.

These similarities and differences are confusing, as well as the taxonomy of checkered, grizzled and marbled skippers. To resolve some of these confusions, we set out to investigate a phylogenetic group consisting of four closely related and exclusively Old World genera *Spialia*, *Gomalia* Moore, 1879, *Carcharodus* and *Muschampia* that we unite in a subtribe Carcharodina Verity, 1940. These genera have received significant attention in literature (Cock, 2016; Coutsis, 2016; de Jong, 1974a, 1974b, 1977, 1978; Devyatkin, 1991; Evans, 1937, 1949; Zhdanko, 1992), including some more recent developments based on molecular studies and description of new cryptic species (Hernandez-Roldan et al., 2016). The four genera have been distinguished largely by appearance of adults and their wing patters and shapes (Evans, 1949). Namely, *Carcharodus* and *Gomalia* both have marbled appearance and hyaline (not opaque) pale spots (if present) near forewing apex and in the discal cell. *Spialia* and *Muschampia* are white-spotted and the spots are opaque. Hindwing is crenulate in *Carcharodus* and *Muschampia*, but more evenly rounded in *Spialia* and *Gomalia*. The outer hindwing margin is somewhat wavy and produced at vein 1A+2A in *Gomalia*, which also differs from the other three genera by uncheckered (or indistinctly checkered) fringes. Furthermore, *Spialia* differs from *Muschampia* by the central pale spot in discal cell on dorsal forewing positioned before the origin of vein CuA_1_ and the pale spot in space CuA_1_-CuA_2_ being in the middle between the discal cell spot and the spot in cell M_3_-CuA_1_, or closer to the latter. In *Muschampia*, the central pale spot in discal cell on dorsal forewing is usually centered around the origin of vein CuA_1_, and if not, then it overlaps with the CuA_1_-CuA_2_ cell spot, which is closer to the discal cell spot than to the spot in M_3_-CuA_1_ cell. This relative simplicity in the genus identification based purely on appearance undoubtedly contributed to the widespread use of Carcharodina classification into these four genera. However, significant variation in genitalic morphology within *Carcharodus* and *Spialia* has been documented (Coutsis, 2016; de Jong, 1974a, 1974b, 1978) suggesting taxonomic complications.

In addition to adults, life histories and immature stages of most Carcharodina species have been documented in detail. Caterpillars of many species feel on Malvaceae and Lamiaceae, however, new foodplants are being discovered. For instance, even for one of the best-studies species, a classic Malvaceae feeder *Carcharodus alceae* (Esper, [1780]) and type species of its genus, which in turn is the type genus of its tribe, Euphorbiaceae were recently discovered as foodplants (Benyamini, 2005). Other plant families have also been used by some species, e.g. Rosaceae, Convolvulaceae, Tiliaceae, Bignoniaceae, and Sterculiaceae (Henning, Henning, Joannou, & Woodhall, 1997; Hernandez-Roldan et al., 2016; Larsen, 1991; Tuzov, 1997). Immature stages of Carcharodina are similar in appearance among species, but some species-specific characters have been discussed by Cock (2016). Caterpillars are covered in short setae, are rather stout and with round dark heads. Most species have brown to purplish-colored caterpillars, with black or yellow spots, however some may be greener or darker to almost back, and *Gomalia* is nearly white. Caterpillars are frequently characterized by a dark collar with several yellow spots (Cock, 2016). The differences between immature stages have not been in good agreement with the current breakdown of species into genera suggesting finer splits. However, *Gomalia* caterpillar is quite recognizable in appearance, being slimmer and paler than others, with thin black collar and more angular head capsule (Cock, 2016).

To better understand phylogenetic relationships and taxonomy of Carcharodina—a challenge from purely morphological perspective—we obtained and analyzed whole genome shotgun DNA sequence reads of representative species, including several primary type specimens (Table S1). The results were mostly in agreement with what has been known about this group of close relatives. However, DNA brings several surprising results: two new genera formed by species previously placed in *Spialia* (de Jong, 1978), and uniqueness of “*Muschampia*” *cribrellum* (Eversmann, 1841), which is not monophyletic with *Muschampia* and instead forms a monotypic genus sister to *Gomalia*. Moreover, wing pattern similarities confused researchers who placed in *Carcharodus* a number of species that actually belong to *Muschampia*. Here, we correct these problems and some others.

## Materials and Methods

We selected 53 out of 67 species from the genera *Spialia*, *Gomalia*, *Carcharodus* and *Muschampia* including representatives of all available genus group names. In addition, we used 8 species from 5 closely related genera as outgroups. One specimen per species was included in the analysis (Table S1). Bodies of freshly collected specimens were stored in RNAlater, and their wings and genitalia dried and kept in envelopes to address possible misidentification issues later. DNA was extracted from a piece of tissue of these specimens. For specimens in museum collections, DNA was extracted either from abdomen or from a leg. Abdomen was gently pushed from above and below (while watching for the legs not to be damaged) until it cracks off and placed in DNA extraction buffer. After extraction (see below), abdomen was transferred to 10% KOH solution and genitalia were dissected in a standard manner. A leg was used for primary type specimens. A leg was removed from a specimen using fine tweezers and placed in a plastic tube. Tweezers were wiped with clean paper tissue after each sample was taken.

DNA was extracted from legs (and abdomens) non-destructively using Macherey-Nagel (MN) reagents. 70 μl buffer T1 and 10 μl protK were simply added to the tube without crushing the leg, and the mixture was incubated at 57°C for 24 hours. Then, 80 μl buffer B3 was added and incubation continued for 2 hours, after which 85 μl of absolute EtOH was added and thoroughly mixed. The resulting liquid was transferred to a different tube and DNA extraction continued according to MN protocol (https://www.mn-net.com/Portals/8/attachments/Redakteure_Bio/Protocols/Genomic%20DNA/UM_gDNATissueXS.pdf), leaving the leg intact. About 70% of DNA was used to construct mate-pair libraries according to our published protocols (Cong et al., 2017).

The libraries were sequenced for 150 bp from both ends on Illumina HiSeq x10 at GENEWIZ. The resulting reads were matched using Diamond (Buchfink, Xie, & Huson, 2015) to the exons of the reference genome of *Cecropterus lyciades* (Geyer, 1832) we have obtained previously (Shen, Cong, Borek, Otwinowski, & Grishin, 2017), exons assembled and aligned to other Hesperiidae genomes we have obtained using the same methods. Coding regions of mitochondrial genome (including the COI barcode) were assembled similarly. Exons expected to be from the Z chromosome were predicted assuming similar syntenic arrangement with *Heliconius* (Heliconius Genome Consortium, 2012). Phylogenetic trees were generated from 3 sets of exons: whole nuclear genome, whole mitochondrial genome and Z-chromosome using RAxML-NG (Kozlov, Darriba, Flouri, Morel, & Stamatakis, 2018) with default parameters (-m GTRGAMMA). PhyML (Guindon et al., 2010) was used to construct the COI barcode tree. The trees were rooted with the sequences of *Noctuana* E. Bell, 1937 and *Windia* H. Freeman, 1969 and 3 other species were used as more distant outgroups (see Table S1 for specimen data). Additional details of methods can be found in the SI Appendix to our recent publication (Li et al., 2019). Sequence data generated in this project were deposited at NCBI as BioProject PRJNA603097. This publication has been registered with ZooBank as http://zoo-bank.org/D934167E-7D2E-41E1-8FFD-24B34C55ABB6.

## Results

### 1. Genomic phylogeny of Carcharodina.

**FIgure 1.**
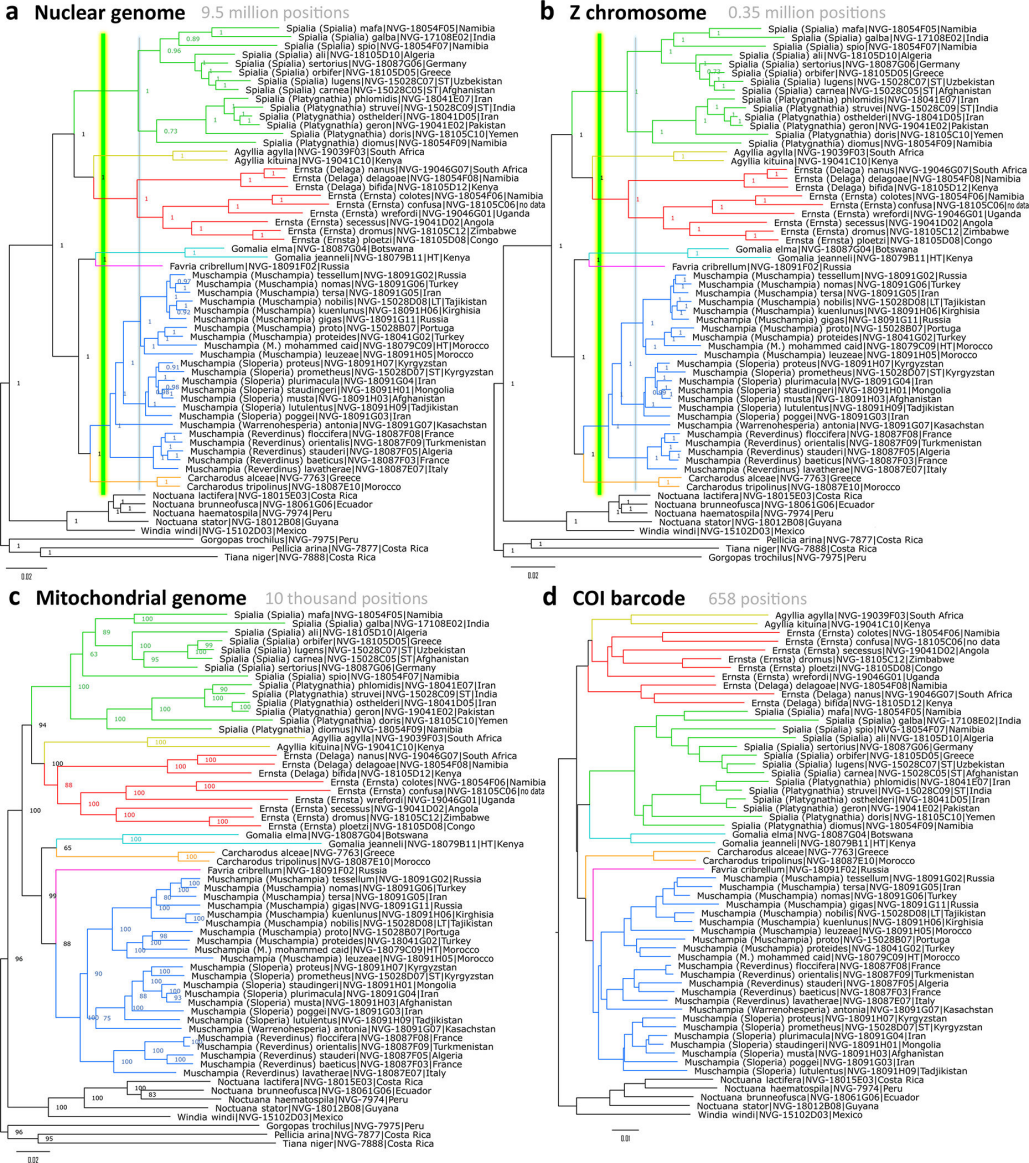
Phylogenetic trees of Carcharodina. The trees are constructed from nucleotide sequences of protein-coding regions from: **a.** nuclear genome; **b.** Z-chromosome; **c.** mitochondrial genome. Statistical significance values are shown by each node. **d.** COI barcode distance diagram is shown to emphasize on close relationships within Carcharodina. In panels a) and b), vertical green (yellow-shaded) line defines genera: each clade crossed by the line is a genus in our treatment; vertical thin gray (blue-shaded) line defines subgenera. Branches in Carcharodina are colored by genus.

We obtained whole genome shotgun sequence reads for 53 representative species of Carcharodina. The lengths of resulting genomic regions were: 9,542,074 +/−3,401,949; Z-chromosome 352,545 +/−136,538; mitogenomes 10,417 +/−1,533. We considered Z-chromosome separately. Butterfly males carry two copies of Z, and females possess Z and W. In Z, recombination is reduced to half of that in autosomes, and sexual selection acts differently on genes encoded by it. Thus the analysis of genes encoded by the Z chromosome may provide additional information about species evolution. Comparison of the phylogenetic trees constructed from nuclear genome, Z chromosome and mitogenome yielded the same conclusions, although only nuclear genomic DNA trees were statistically confident at most nodes (Fig. 1).

First, species placed in the 4 Old World genera *Spialia*, *Gomalia*, *Carcharodus* and *Muschampia* are monophyletic and form a clade well separated from the outgroups. Thus, it is meaningful to assign this clade a rank of subtribe (Carcharodina). Second, instead of splitting into 4 clades according to the original genus names, the group forms 7 clades shown in different colors in Fig. 1. These clades are defined by a green line crossing the tree, the idea used in other works (Li et al., 2019; Talavera, Lukhtanov, Pierce, & Vila, 2012)), see Discussion below for details. Notably, *Spialia* is split into 3 clades, two of which are not even sisters in the COI barcode dendrogram. The separation between the three clades suggests that they represent three genera, two of which do not have names and are described here. In addition, one of these clades partitions into two subclades, one of which is described as a subgenus. Description of these three new taxa follows.

#### *Ernsta* grishin, gen. n.

http://zoobank.org/8301DAE5-F4D8-4EE8-BFDC-BFF4BCE2A8E9

#### Type species:

**FIgure 2.**
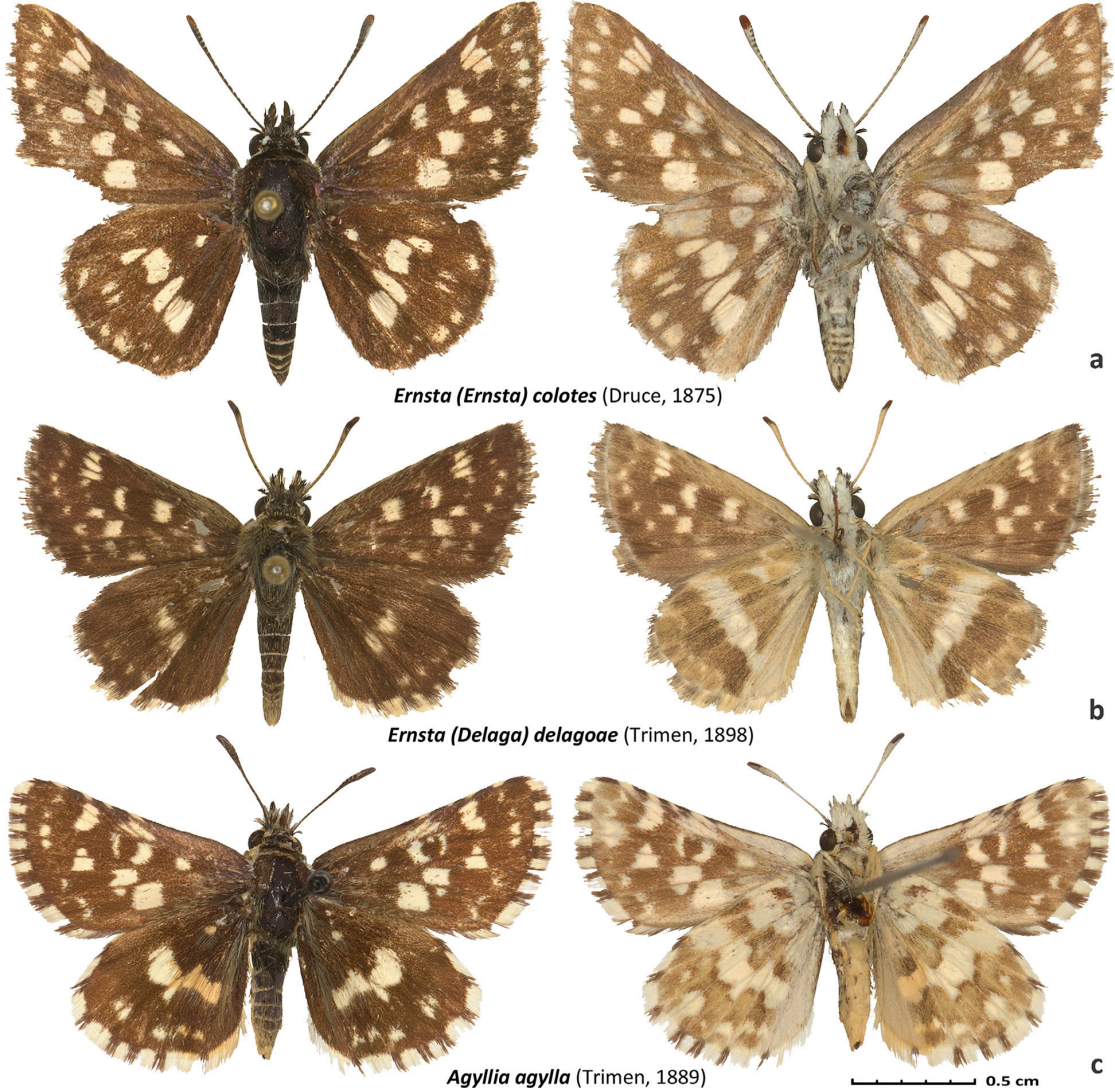
Specimens of *Ernsta* and *Agyllia*. Dorsal and ventral views are on the left and right, respectively. DNA sample IDs are given, other data are in the Tab. S1: **a**. *Ernsta colotes* the type species of the genus *Ernsta* gen. n., NVG-18054F06; **b**. *Ernsta delagoae*, the type species of the subgenus *Delaga* subgen. n., NVG-18054F08; c. *Agyllia agylla*, the type species of the genus *Agyllia* gen. n., NVG-19039F03.

*Pyrgus colotes* Druce, 1875 (Fig. 2a).

#### Diagnosis.

Morphologically similar to *Spialia* Swinhoe, 1912, where these species were placed previously. Keys to 5, 15, 21 (exclude antithesis of 25), or thesis 11 in de Jong (1978: 28 & 30), constituting his *colotes*, *delagoae*, and *dromus* species groups. Differs from its relatives by the following characters. Ventral hindwing with straight median white band not separated into sports, i.e., white spot in cell RS-M_1_ (space 6) joins central spot (discal cell) to the outer (and not inner) spot in cell Sc+R_1_-RS (space 7), but in many species of *Spialia* this band either broken into spots or directed basad at costa. While *Spialia* species lack costal fold in males, some *Ernsta* species have costal fold (*colotes* species group). Species with costal fold are in addition characterized by the central white discal cell spot on dorsal forewing not closer to discocellular spot than to the basal cell spot and no two white spots are present above over the central cell spot (to distinguish from *asterodia* species group of de Jong (1978) that does not belong to *Ernsta*) and hindwing submarginal pale spots in cells M_1_-M_2_ & M_2_-M_3_ (spaces 4 & 5) offset basad from the rest of the submarginal spots. Species without costal fold either lack the basal white spots in discal cell on dorsal forewing, however, some white scales along cubital vein may be present forming a narrow streak (the *delagoae* species group), or on dorsal forewing in CuA_2_-1A+2A cell (space 1B) the outer lower median spot absent and inner lower median spot not smaller than the outer upper median spot (*dromus* species group). In male genitalia, uncus not deeply incised, gnathos dorsally joined to tegumen, if gnathos free (in some species from the *delagoae* group), then coecum of aedeagus shortened or absent. In DNA COI barcode region, a combination of the following base pairs is diagnostic: A46T, C278T, T280A, T282T (not C), T301T (not C), T349A, G353G (not T), A481A (not T or C), and 529(not T).

#### Derivation of the name.

The name is a feminine noun in the nominative singular. It honors Ernst Brockmann of Lich, Germany and his unstoppable passion for Hesperiidae in general and the Grizzled skippers in particular. Without his enthusiasm, help, encouragement and specimens this study would not be accomplished.

#### Species included:

Encompasses *delagoae*, *colotes*, and *dromus* species groups, as they were defined by de Jong (1978). Full species list is given below. These are mostly African species, only three of which (*E. colotes*, *E. mangana*, and *E. bifida*) cross the Red Sea into the southern corner of the Arabian Peninsula, and one (*E. zebra*) is recorded from the northwestern Himalayas.

The phylogenetic trees (Fig. 1) suggest that this genus has split into two groups: one contains the type species of the genus, and the other one is named here as a subgenus.

#### *Delaga* grishin, subgen. n.

http://zoobank.org/A5431ACA-C253-4414-AE23-97A320D45D4D

#### Type species:

*Pyrgus delagoae* Trimen, 1898 (Fig. 2b).

#### Diagnosis.

Keys to 15 in de Jong (1978: 30), constituting his *delagoae* species group. Morphologically differs from other species in the genus by the following characters. Forewing dorsal white spots at the base of CuA_2_-1A+2A cell (space 1B) absent, and the spot at the base of the discal cell is absent in most species (some white scales along cubital vein may be present forming a narrow streak). Ventral hindwing with a straight median white band, i.e., a white spot in cell RS-M_1_ (space 6) joins the central spot (discal cell) to the outer (and not inner) spot in cell Sc+R_1_-RS (space 7). In male genitalia, coecum of aedeagus shortened or absent; valva with the costal process and harpe (=cucullus) lacks a fold covering the costal process, or the fold is small. In DNA COI barcode region, a combination of the following base pairs is diagnostic: T19A, T22A, T70A, T374G, and T646C.

#### Derivation of the name.

The name is a feminine noun in the nominative singular derived from the name of the type species.

#### Species included:

Encompasses *delagoae* species groups, as it was defined by de Jong (1978). Full species list is given below.

In addition to *Ernsta*, the phylogenetic trees (Fig. 1) suggest a second new genus, which while being monophyletic with *Ernsta* is prominently different from it.

#### *Agyllia* grishin, gen. n.

http://zoobank.org/095B9432-5CCE-4CBF-8EB6-B9711FDABA25

#### Type species:

*Pyrgus agylla* Trimen, 1889 (Fig. 2c).

#### Diagnosis.

Keys to 2 in de Jong (1978: 28), constituting his *asterodia* species group. Morphologically differs from close relatives by the following characters. Out of three spots in forewing discal cell, rectangular middle spot (the largest) closer to streak-like spot at distal end of cell than to well-developed and rounded basal spot; no dorsal white spots at base of CuA_2_-1A+2A cell (space 1B). Ventral hindwing with a straight median white band, i.e., a white spot in cell Rs-M_1_ (space 6) joins the central spot (discal cell) to the outer (and not inner) spot in cell Sc+R_1_-Rs (space 7). In male genitalia, uncus deeply incised; valva with large costal process and harpe (=cucullus) lacks a fold covering the costal process. In DNA COI barcode region, a combination of the following base pairs is diagnostic: A307T, A352T, T364C, C401T, T403A, T500C, and A502T.

#### Derivation of the name.

The name is a feminine noun in the nominative singular derived from the name of the type species.

#### Species included:

Encompasses *asterodia* species groups as it was defined by de Jong (1978). Full species list is given below.

Second, we observe that *Carcharodus* is not monophyletic. Only one species, *Carcharodus tripolina* (Verity, 1925) groups with the type species of the genus *Carcharodus alceae* (Esper, 1780). These results are consistent with the recent treatment by Coutsis (2016), who placed all other *Carcharodus* species in *Reverdinus* Ragusa, 1919. In our trees, *Reverdinus* is in the same cluster with *Muschampia* Tutt, 1906 and the branch length separating *Reverdinus* from other *Muschampia* is not significantly larger than the branch lengths separating *Muschampia* species from each other. Thus, we consider *Reverdinus* Ragusa, 1919 to be a subgenus of *Muschampia*. Additionally, we see that genus names previously given to various groups currently placed in *Muschampia* indeed denote monophyletic groups within the genus and we suggest to treat these groups as subgenera: *Warrenohesperia* Strand, 1928, *Sloperia* Tutt, 1906 and *Tuttia* Warren, 1926 (Fig. 1).

Third, we find that “*Muschampia*” *cribrellum* (Eversmann, 1841), the type species of the genus *Favria* Tutt, 1906 is not monophyletic with *Muschampia*. Instead, it is a confident sister of *Gomalia* in nuclear genome trees (Fig. 1ab). Its phylogenetic position is not very strongly supported in the mitogenome tree (88% bootstrap, Fig. 1c), but it is well-separated from *Muschampia*. This species has been a puzzle and is uniquely characterized by spined mid-tibiae. Therefore we reinstate *Favria* as a valid genus, currently monotypic.

**FIgure 3.**
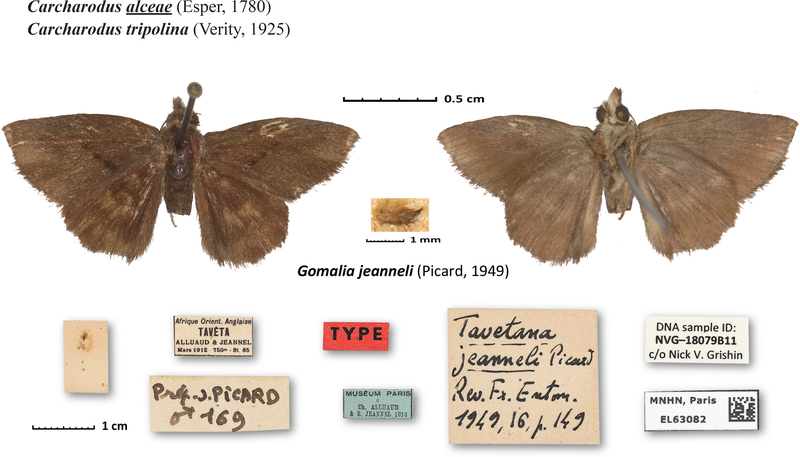
*Gomalia jeanneli* holotype. The specimen is in the Muséum National d’Histoire Naturelle, Paris, France. Dorsal and ventral views are on the left and right, respectively. The largest scale bar refers to the specimen, labels are shown at 1/3 of specimen size and genitalia enlarged twice compared to the specimen. DNA sample ID NVG-18079B11.

Fourth, we see that the holotype of *Tavetana jeanneli* Picard, 1949 (Fig. 3) is not a dark form of *Gomalia elma* (Trimen, 1862) as currently considered, but a *Gomalia* species well removed from it. COI barcodes of the two species differ by nearly 7% (45 base pairs). Moreover, the differences in genitalia of the Indian *Gomalia elma albofasciata* Moore, 1879 (see plate 23, D2 in Evans, 1949) and the African nominal subspecies (plate 13 in Evans, 1937) argue for the species status of the Indian taxon. Most notably, ampulla of male genitalic valva is expanded in *C. albofasciata* compared to *C. elma*, in which costa smoothly transitions to a tooth-like ending of harpe.

Furthermore, we elevate to species *Spialia lugens* (Staudinger, 1886) and *Spialia carnea* (Reverdin, 1927) formerly considered subspecies of *Spialia orbifer* (Hübner, [1823]). Sequencing of *S. lugens* and *S. carnea* type specimens in the Berlin Museum für Naturkunde reveals 2.2%−3.2% difference in COI barcode from nominotypical populations of *S. orbifer*. Distinct barcodes combined with the differences in facies suggest species-level status for these taxa. *Spialia lugens* differs from the two other species by the larger size, darker wing above with faint or absent submarginal sports, rarely, and mostly in females, better developed (de Jong, 1978). *S. carnea* is characterized by warm reddish to brown-yellow color of hindwing below and reduced submarginal spots on hindwing below in cells M_1_-M_2_ and M_2_-M_3_.

Finally, difference in male genitalia, notably the shape of uncus (de Jong, 1978; Evans, 1937), suggest that *Ernsta bifida* (Higgins, 1924), a species distinct from *Ernsta zebra* (Butler, 1888) and not its subspecies. Taken together, the data we obtained suggest the following taxonomic arrangement of the subtribe Carcharodina.

#### Taxonomic arrangement of the subtribe Carcharodina.

Based on our analysis, the list of species arranged into genera and subgenera is given below. Synonymic names are included for genera and subgenera. Names treated as synonyms (genera and names of type species that are considered to be synonyms) are preceded by “=”: not followed by daggers are subjective junior synonyms; † objective junior synonyms; ‡ unavailable names (such as homonyms and nomina nuda); “preocc.” indicates preoccupied, the taxonomic order (all insects) of the senior name is shown in brackets. Synonyms are attributed to subgenera. Type species (TS) for genera and subgenera are listed and underlined. For type species that are considered to be synonyms, valid names are shown in parenthesis. For valid genera and subgenera (not their synonyms), names of the type species or names which type species are considered to be synonyms of, are underlined in the list. Subspecies names are not listed pending further studies.

##### Subtribe Carcharodina Verity, 1940

###### *Spialia* Swinhoe, 1912; TS: *galba* Fabricius

Subgenus *Spialia* Swinhoe, 1912; TS: *galba* Fabricius

=‡*Powellia* Tutt, 1906 (preoc. Maskell, 1879 [Hemiptera]); TS: =‡*sao* Hübner, 1800 (*sertorius* Hoffmansegg)

=*Neospialia* Koçak, 1989; TS: =‡*sao* Hübner, 1800 (*sertorius* Hoffmansegg)

***Spialia mafa*** (Trimen, 1870)
***Spialia*** ***galba*** (Fabricius, 1793)
***Spialia spio*** (Linnaeus, 1764)
***Spialia ali*** Oberthür, 1881
***Spialia therapne*** (Rambur, 1832)
***Spialia sertorius*** (Hoffmannsegg, 1804)
***Spialia rosae*** Hernández-Roldán, Dapporto, Dincă, Vicente & Vila, 2016
***Spialia orbifer*** (Hübner, [1823])
***Spialia lugens*** (Staudinger, 1886); new status, was a subspecies of *S. orbifer*
***Spialia carnea*** (Reverdin, 1927); new status, was a subspecies of *S. orbifer*

Subgenus *Platygnathia* Picard, 1948; stat. rev., was a synonym of *Spialia*; TS: *phlomidis* Herrich-Schäffer

***Spialia*** ***phlomidis*** (Herrich-Schäffer, [1845])
***Spialia struvei*** (Püngeler, 1914)
***Spialia fetida*** Zhdanko, 1992
***Spialia irida*** Zhdanko, 1993
***Spialia osthelderi*** (Pfeiffer, 1932)
***Spialia geron*** (Watson, 1893)
***Spialia doris*** (Walker, 1870)
***Spialia diomus*** (Hopffer, 1855)
***Spialia ferax*** (Wallengren, 1863)

###### *Agyllia* grishin, new genus; TS: *agylla* Trimen

***Agyllia asterodia*** (Trimen, 1864); new combination, was in *Spialia*
***Agyllia*** ***agylla*** (Trimen, 1889); new combination, was in *Spialia*
***Agyllia kituina*** (Karsch, 1896); new combination, was in *Spialia*

###### *Ernsta* grishin, new genus; TS: *colotes* Druce

Subgenus *Delaga* Grishin, new subgenus; TS: *delagoae* Trimen

***Ernsta mangana*** (Rebel, 1899); new combination, was in *Spialia*
***Ernsta nanus*** (Trimen, 1889); new combination, was in *Spialia*
***Ernsta delagoae*** (Trimen, 1898); new combination, was in *Spialia*
***Ernsta zebra*** (Butler, 1888); new combination, was in *Spialia*
***Ernsta bifida*** (Higgins, 1924); new combination, reinstated status, was a subspecies of *Spialia zebra*
***Ernsta sataspes*** (Trimen, 1864); new combination, was in *Spialia*
***Ernsta depauperata*** (Strand, 1911); new combination, was in *Spialia*

Subgenus *Ernsta* Grishin; TS: *colotes* Druce

***Ernsta*** ***colotes*** (Druce, 1875); new combination, was in *Spialia*
***Ernsta confusa*** (Higgins, 1924); new combination, was in *Spialia*
***Ernsta wrefordi*** (Evans, 1951); new combination, was in *Spialia*
***Ernsta paula*** (Higgins, 1924); new combination, was in *Spialia*
***Ernsta secessus*** (Trimen, 1891); new combination, was in *Spialia*
***Ernsta dromus*** (Plötz, 1884); new combination, was in *Spialia*
***Ernsta ploetzi*** (Aurivillius, 1891); new combination, was in *Spialia*

###### *Gomalia* Moore, 1879; TS: *albofasciata* Moore

=*Tavetana* Picard, 1949; TS: *jeanneli* Picard

***Gomalia elma*** (Trimen, 1862)
***Gomalia jeanneli*** (Picard, 1949); stat. rev., was a synonym of *G. elma*
***Gomalia*** ***albofasciata*** Moore, 1879; stat. rev., was a subspecies of *G. elma*

###### *Favria* Tutt, 1906; stat. rev., was a synonym of *Muschampia*; TS: *cribrellum* eversmann

***Favria*** ***cribrellum*** (Eversmann, 1841)

###### *Muschampia* Tutt, 1906; TS: *proto* Ochsenheimer

Subgenus *Muschampia* Tutt, 1906; TS: *proto* Ochsenheimer

=*Tuttia* Warren, 1926; TS: *tessellum* Hübner

***Muschampia tessellum*** (Hübner, [1800–1803])
***Muschampia nomas*** (Lederer, 1855)
***Muschampia tersa*** Evans, 1949
***Muschampia nobilis*** (Staudinger, 1882)
***Muschampia kuenlunus*** (Grum-Grshimailo, 1893)
***Muschampia protheon*** (Rambur, 1858)
***Muschampia gigas*** (Bremer, 1864)
***Muschampia*** ***proto*** Ochsenheimer, 1808
***Muschampia proteides*** (F.Wagner,1929)
***Muschampia mohammed*** (Oberthür, 1887)
***Muschampia leuzeae*** (Oberthür, 1881)

Subgenus *Sloperia* Tutt, 1906; stat. rev., was a synonym of *Muschampia*; TS: *poggei* Lederer

=*Reverdinia* Warren, 1926; TS: *staudingeri* Speyer

***Muschampia proteus*** (Staudinger, 1886)
***Muschampia prometheus*** (Grum-Grshimailo, 1890)
***Muschampia plurimacula*** (Christoph, 1893)
***Muschampia staudingeri*** (Speyer, 1879)
***Muschampia musta*** Evans, 1949
***Muschampia lutulentus*** (Grum-Grshimailo, 1887)
***Muschampia*** ***poggei*** (Lederer, 1858)

Subgenus *Warrenohesperia* Strand, 1928; stat. rev., was a synonym of *Muschampia*; TS: *antonia* Speyer

=‡*Ramburia* Warren, 1926 (preoc. Robineau-Desvoidy, 1851 [Diptera]); TS: *antonia* Speyer

***Muschampia*** ***antonia*** (Speyer, 1879)

Subgenus *Reverdinus* Ragusa, 1919; stat. rev., new placement, was a synonym of *Carcharodus*; TS: =‡*altheae* Hübner, [1800–1803] (*floccifera* Zeller)

=*Lavatheria* Verity, 1940; new placement, was a synonym of *Carcharodus*; TS: *lavatherae* Esper

***Muschampia*** ***floccifera*** (Zeller, 1847); new combination, was in *Carcharodus*
***Muschampia orientalis*** (Reverdin, 1913); new combination, was in *Carcharodus*
***Muschampia dravira*** (Moore, [1875]); new combination, was in *Carcharodus*
***Muschampia stauderi*** (Reverdin, 1913); new combination, was in *Carcharodus*
***Muschampia baeticus*** (Rambur, 1840); new combination, was in *Carcharodus*
***Muschampia lavatherae*** (Esper, 1783); new combination, was in *Carcharodus*

###### *Carcharodus* Hübner, [1819]; TS: *alceae* esper

=†*Syrichtus* Boisduval, [1834]; TS: *alceae* Esper

=†*Spilolhyrus* Duponchel, 1835; TS: *alceae* Esper

***Carcharodus*** ***alceae*** (Esper, 1780)
***Carcharodus tripolina*** (Verity, 1925)

### Identification key to genera of Carcharodina.

The key provides phenotypic characters for all Carcharodina genera to aid their identification.

1. Hindwing outer margin evenly rounded without crenulation. Fringes prominently checkered. Wings white-spotted (checkered appearance, i.e. dark-brown background with many opaque pale spots): central pale spot in discal cell on dorsal forewing positioned before the origin of vein CuA_1_ and the pale spot in space CuA_1_-CuA_2_ positioned in the middle between the discal cell spot and the spot in cell M_3_-CuA_1_, or closer to the latter. . . . . . . . . . . . . . . . . . . . . . . . . . . . . . . . . . . . . . . . . . . . . . . . . . . . . . . . 2

- Hindwing more or less crenulate or fringes uncheckered and hindwing slightly produced at vein 1A+2A. Wings marbled and with hyaline spots, if white-spotted, then central pale spot in discal cell on dorsal forewing usually centered around the origin of vein CuA_1_, and if not, then it overlaps with the CuA_1_-CuA_2_ cell spot, which is closer to the discal cell spot than to the spot in M_3_-CuA_1_ cell. . . . . . . . . . . . . . . . . . . . . . . . . . . . . . . . . . . . . . . . . . . . . . . . . . . . . . . . . . . . . . . . . . . . . . . . . . . . . . . . . . . . . . . . . 4

- Out of three spots in forewing discal cell, rectangular middle spot (the largest) closer to streak-like spot at distal end of cell than to well-developed and rounded basal spot. Male with costal fold. Uncus deeply incised. . . . . . . . . . . . . . . . . . . ***Agyllia*** gen. n.

- Out of three spots in forewing discal cell, rectangular middle spot (the largest) not closer to streak-like spot at distal end of cell than to basal spot, or basal spot absent. Male with or without costal fold. Uncus not deeply incised. . . . . . . . . . . . . . . . . . . . . 3

- Ventral hindwing with straight median white band not separated into sports, i.e., white spot in cell RS-M_1_ joins central spot (discal cell) to the outer (and not inner) spot in cell Sc+R_1_-RS. Hindwing submarginal pale spots in cells M_1_-M_2_ & M_2_-M_3_ offset basad from the rest of the submarginal spots in species with costal fold. Species without costal fold either lack basal white spots in discal cell on dorsal forewing (some white scales along cubital vein may be present forming a narrow streak), or on dorsal forewing in CuA_2_-1A+2A cell the outer lower median spot absent and inner lower median spot forming a bar with inner upper median spot, larger than the outer upper median spot. Gnathos dorsally joined to tegumen, if gnathos free, then coecum of aedeagus shortened or absent. . . . . . . . . . . . . . . . . . . . . . . . . . . . . . . . . . . . . . . . . . . . . . . . . . . . . . . . . . . . . . . . . .***Ernsta*** gen. n.

- Ventral hindwing median white band frequently broken into spots or if not, then usually directed basad at costa. Hindwing submarginal pale spots in cells M_1_-M_2_ & M_2_-M_3_ in line with other submarginal spots or absent. In species with straight entire median white ventral hindwing band (similar to *Ernsta* gen. n.), basal white spots in discal cell on dorsal forewing present and in CuA_2_-1A+2A cell inner upper median spot absent, outer upper median spot well developed, nearly the same size as inner lower median spot. Male without costal fold. Gnathos not joined to tegumen, aedeagus typically with coecum. . . . . . . ***Spialia***

- Fringes not checkered or indistinctly checkered. Hindwing outer margin wavy and slightly produced at vein 1A+2A. Mid-tibiae without a row of spines. Wings marbled and usually with hyaline spots. Caterpillar almost white, more elongated, foodplants Malvaceae. . . . . . . . . . . . . . . . . . . . . . . . . . . . . . . . . . . . . . . . . . . . . . . . . . . . . . . . . . . . . . . . . . . . . . . . . . . . . . . . . . . . . ***Gomalia***

- Fringes prominently checkered. Hindwing more or less crenulate. Caterpillar darker and stouter. . . . . . . . . . . . . . . . . . . . . . . 5

- Mid-tibiae with a row of spines. Costa of valva with broad serrated process directed ventrad. Wings white-spotted. Single species currently included in this genus exhibits a nearly perfect, but apparently convergent, similarity in wing pattern with *Muschampia tessellum* (Hübner, [1800–1803]), differing by the subapical forewing white bar that almost always consists of 4 to 5 (instead of 3) spots. . . . . . . . . . . . . . . . . . . . . . . . . . . . . . . . . . . . . . . . . . . . . . . . . . . . . . . . . . . . . . . . . . . . . . . . . . . . .***Favria***

- Mid-tibiae smooth, without a row of spines. Costa of valva without such process. Wings white-spotted or marbled and with hyaline spots. . . . . . . . . . . . . . . . . . . . . . . . . . . . . . . . . . . . . . . . . . . . . . . . . . . . . . . . . . . . . . . . . . . . . . . . . . . . . . . . . . . . . . . . . 6

- Aedeagus thin. Uncus not longer than tegumen. Wings white-spotted, if marbled, then valva longer than wide and forewing pale bar defining the end of discal cell and central hyaline spots well-developed. Caterpillar foodplants (where known) Lamiaceae. . . . . . . . . . . . . . . . . . . . . . . . . . . . . . . . . . . . . . . . . . . . . . . . . . . . . . . . . . . . . . . . . . . . . . . . . . . . . . . . . . . . . . . . . . ***Muschampia***

- Aedeagus very broad, expanded distally. Uncus longer than tegumen. Valva nearly as long as wide. Wings marbled and with hyaline spots, forewing pale bar defining the end of discal cell absent or inconspicuous and central hyaline spots smaller. Caterpillar foodplants Malvaceae and Euphorbiaceae. . . . . . . . . . . . . . . . . . . . . . . . . . . . . . . . . . . . . . . . . . . . . . . . . . ***Carcharodus***

## Discussion

We find the genomic perspective on the subtribe Carcharodina to be quite insightful. Complementary to morphological analysis, it confidently reveals new phylogenetic affinities and uniqueness of certain phylogenetic lineages. Many millions of base pairs in nuclear genome gene coding regions result in highly confident phylogenies and reveal well-supported monophyletic groups. We use these groups to refine the classification of Carcharodina and instead of 4 genera used previously, we utilize 7 (see the taxonomic list above). While our genera form very confident clades (Fig. 1), other equally confident clades could have been chosen. Currently, there is no agreed upon objective criteria for defining a genus. It has been argued that a cut through phylogenetic tree may define genera consistently (Li et al., 2019; Talavera et al., 2012). However, exact position of such cut in a tree remains undefined. We utilized this approach to define genera with a cut (green line in Fig. 1ab) in a way that preserves currently defined genera. However, it is possible to move the position of the cut in either direction.

Pushing the cut back in time, we will lose, *Agylla* to *Ernsta*, *Favria* to *Gomalia*, and *Muschampia* to *Carcharodus*. It is not clear that such treatment is better. First, *Gomalia* and *Favria* are phenotypically different, not giving immediate confidence in their unification. Second, branches in the tree that support each of the three pairs *Ernsta* + *Agylla*, *Gomalia* + *Favria*, and *Carcharodus* + *Muschampia* are shorter than the branches supporting each of these six genera individually. Thus, the combined genera are less prominent, and therefore are possibly less confident and less identifiable groups. Third, we will lose the name *Muschampia*, a genus traditionally used for decades.

Cutting the tree even further back in time results in just two genera: *Carcharodus* (including *Gomalia*, *Muschampia*, and *Favria*) and *Spialia* (including *Ernsta* and *Agylla*). This would be a lumping treatment that nevertheless is appealing. Although the two genera are less prominent than our 7 (*Spialia*, *Agylla*, *Ernsta*, *Gomalia*, *Favria*, *Muschampia*, and *Carcharodus*), they are better defined than 4 (*Spialia*, *Ernsta* [with *Agylla* as its subgenus or synonym: as the first reviser we give priority to *Ernsta*], *Gomalia* [with *Favria* as its subgenus or synonym], and *Carcharodus* [with *Muschampia* as its subgenus or synonym]) and keep former *Spialia* intact. It is also possible to opt for an inconsistent treatment when some genera originated later than others, and a single cut through the tree does not define them. Future usage of these names and research will settle this question.

## Conclusions.

Grizzled and Marbled skippers of the Old Word are abundant and have been studied extensively over the years, likely better than many other groups of Hesperiidae. To our surprise, we found that genomic analysis gives a different perspective on their classification. We detect distinct phylogenetic lineages that we treat as new genera and we define subgenera. We correct phylogenetic placement of some species assigned to a genus they do not belong. We see that adding genomic analysis to the arsenal of taxonomists reveals findings that are not easy to obtain using morphological analysis.

## Supplementary Material

Supplementary file
