# Supplementary material for: A genomic perspective on the taxonomy of the subtribe Carcharodina (Lepidoptera: Hesperiidae: Carcharodini)
Source: Zootaxa. Author manuscript; Available in PMC 2021 Apr 2. (PMC8018707; doi:10.11646/zootaxa.4748.1.10)
Supplement: Supplementary file [file NIHMS1682343-supplement-Supplementary_file.pdf]

Table S1. Data for 53 sequenced Carcharodini and 8 outgroup species

#	DNA Voucher	Taxon name	Type	Sex	Locality, collectors, date	Collection	Collection No.
1	NVG-18054F05	***Spialia (Spialia) mafa***			Namibia, 14-20-Apr-2001	ZMHB	
2	NVG-17108E02	***Spialia (Spialia) galba***		F	India, 5-Oct-1961	LACM	
3	NVG-18054F07	***Spialia (Spialia) spio***			Namibia, 14-18-Apr-2001	ZMHB	
4	NVG-18105D10	***Spialia (Spialia) ali***			Algeria: Djurdjura, Jul-1884	USNM	
5	NVG-18087G06	***Spialia (Spialia) sertorius***			Germany: Kaiserstuhl, Bickensohl, E. Brockmann, 4-6-Jul-1985	Brockmann	
6	NVG-18105D05	***Spialia (Spialia) orbifer***			Greece, 20-Sep-1964	USNM	
7	NVG-15028C07	***Spialia (Spialia) lugens***	ST	M	Uzbekistan: Fergana Region, Margilan, 1884	ZMHB	
8	NVG-15028C05	***Spialia (Spialia) carnea***	ST	M	Afghanistan: Paghman Mtns., Aug prior to 1927	ZMHB	
9	NVG-18041E07	***Spialia (Platygnathia) phlomidis***		M	Iran: Shah Kuh, Till Abah, 1600 m, 36.867, 55.417, J. Klir, 24-May-2008	Brockmann	
10	NVG-15028C09	***Spialia (Platygnathia) struvei***	ST	M	India: E. Tian-Shan, Mountains north of Barkul, Ruckbeil, 1908	ZMHB	
11	NVG-18041D05	***Spialia (Platygnathia) osthelderi***		M	Iran: Isfahan, 20 km S Isfahan, 1700-1800 m, W. Eckweiler, 29-Jun-2003	Brockmann	
12	NVG-19041E02	***Spialia (Platygnathia) geron***			Pakistan: Balochistan, Kuhan, 16-Jun-1928	AMNH	AMNH_IZC 00337776
13	NVG-18105C10	***Spialia (Platygnathia) doris***			Yemen: Aden, Janson, 1907	USNM	
14	NVG-18054F09	***Spialia (Platygnathia) ferax***			Namibia, 24-29-Mar-2002	ZMHB	
15	NVG-19039F03	***Agyllia agylla***		F	South Africa: Bloemfontein, Sep-1914	AMNH	AMNH_IZC 00337693
16	NVG-19041C10	***Agyllia kituina***			Kenya: Garissa District, Ukazzi hill, Garissa Rd., van Someren, Dec-1948	AMNH	AMNH_IZC 00337760
17	NVG-19046G07	***Ernsta (Delaga) nanus***			South Africa: Bloemfontein, 10-Feb-1927	AMNH	AMNH_IZC 00346642
18	NVG-18054F08	***Ernsta (Delaga) delagoae***		M	Namibia, 24-29-Mar-2002	ZMHB	
19	NVG-18105D12	***Ernsta (Delaga) bifida***			Kenya, Jan-1959	USNM	
20	NVG-18054F06	***Ernsta (Ernsta) colotes***		M	Namibia, 14-20-Apr-2001	ZMHB	
21	NVG-18105C06	***Ernsta (Ernsta) confusa***			no data, B. Neumoegen collection, around 1900	USNM	
22	NVG-19046G01	***Ernsta (Ernsta) wrefordi***			Uganda, Jun-1954	AMNH	AMNH_IZC 00346636
23	NVG-19041D02	***Ernsta (Ernsta) secessus***		F	Angola, Aug-1925	AMNH	AMNH_IZC 00337764
24	NVG-18105C12	***Ernsta (Ernsta) dromus***			Zimbabwe: Mutare, Nov-1953	USNM	
25	NVG-18105D08	***Ernsta (Ernsta) ploetzi***			Congo, Mar-1959	USNM	
26	NVG-18087G04	***Gomalia elma***			Botswana: Nata, M. Snizek, 9-14-Jan-1997	Brockmann	
27	NVG-18079B11	***Gomalia jeanneli***	HT	M	Kenya: Taita-Taveta Co., Taveta, 750 m, Alluaud & Jeannel, Mar-1912	MNHP	EL63082
28	NVG-18091F02	***Favria cribrellum***		M	Russia: Ural, 19-Jun-1999	Brockmann	
29	NVG-18091G02	***Muschampia (Muschampia) tessellum***		M	Russia: Tuva, 28-Jun-2002	Brockmann	
30	NVG-18091G06	***Muschampia (Muschampia) nomas***		F	Turkey, 15-17-Jul-1992	Brockmann	
31	NVG-18091G05	***Muschampia (Muschampia) tersa***		M	Iran, 19-Apr-2001	Brockmann	
32	NVG-15028D08	***Muschampia (Muschampia) nobilis***	LT	M	Tajikistan: Farob, 1881	ZMHB	
33	NVG-18091H06	***Muschampia (Muschampia) kuenlunus***			Kyrgyzstan: Naryn, 11-12-Jul-1992	Brockmann	
34	NVG-18091G11	***Muschampia (Muschampia) gigas***			Russia: Primorye, 23-30-Jul-1998	Brockmann	
35	NVG-15028B07	***Muschampia (Muschampia) proto***		F	Portugal: Algarve, around 1900	ZMHB	
36	NVG-18041G02	***Muschampia (Muschampia) proteides***		M	Turkey: Nigde, Demirkazik, 1850 m, L. Bieber, July 2008	Brockmann	
37	NVG-18079C09	***Muschampia (M.) mohammed caid***	HT	M	Morocco: Oulmes, 1-Apr-1922	MNHP	EL63092
38	NVG-18091H05	***Muschampia (Muschampia) leuzeae***			Morocco, 10-Jun-1973	Brockmann	
39	NVG-18091H07	***Muschampia (Sloperia) proteus***			Kyrgyzstan: Alai, 20-25-Jun-1998	Brockmann	
40	NVG-15028D07	***Muschampia (Sloperia) prometheus***	ST		Kyrgyzstan: Trans-Alay Range, Zaalaisky Mtns., 1885	ZMHB	
41	NVG-18091G04	***Muschampia (Sloperia) plurimacula***		M	Iran, 23-May-2013	Brockmann	
42	NVG-18091H01	***Muschampia (Sloperia) staudingeri***		M	Mongolia, 4-Jul-2004	Brockmann	
43	NVG-18091H03	***Muschampia (Sloperia) musta***		M	Afghanistan, 5-Jul-2013	Brockmann	
44	NVG-18091H09	***Muschampia (Sloperia) lutulentus***			Tajikistan, 4-7-Jul-2000	Brockmann	
45	NVG-18091G03	***Muschampia (Sloperia) poggei***		F	Iran, 12-Jun-2000	Brockmann	
46	NVG-18091G07	***Muschampia (Warrenohesperia) antonia***		M	Kazakhstan, 19-23-May-2007	Brockmann	
47	NVG-18087F08	***Muschampia (Reverdinus) floccifera***		M	France, 20-Jul-1995	Brockmann	
48	NVG-18087F09	***Muschampia (Reverdinus) orientalis***		M	Turkmenistan, May-1994	Brockmann	
49	NVG-18087F05	***Muschampia (Reverdinus) stauderi***		M	Algeria, May-1995	Brockmann	
50	NVG-18087F03	***Muschampia (Reverdinus) baeticus***		M	France, 8-Jul-1995	Brockmann	
51	NVG-18087E07	***Muschampia (Reverdinus) lavatherae***		M	Italy, 18-Jun-1991	Brockmann	
52	NVG-7763	***Carcharodus alceae***		M	Greece, 20-Oct-1990	USNM	
53	NVG-18087E10	***Carcharodus tripolinus***		M	Morocco, 10-25-Apr-1989	Brockmann	
54	NVG-18015E03	***Noctuana lactifera***			Costa Rica: ACG, 1150 m, 10.92691, -85.46822, eclosed on 27-Oct-2003	USNM	03-SRNP-22972
55	NVG-18061G06	***Noctuana brunneofusca***			Ecuador, 15-May-1988	USNM	
56	NVG-7974	***Noctuana haematospila***		M	Peru: Cuzco, Cosnipata Road, Mirador, 1700 m, Stephen Kinyon, 2-Feb-2011	USNM	
57	NVG-18012B08	***Noctuana stator***			Guyana, 31-Oct-10-Nov-2000	USNM	
58	NVG-15102D03	***Windia windi***		M	Mexico: Sonora, Rte 16, 8.5 mi W. Rio Yaqui, Douglas Mullins, 26-Aug-1984	USNM	
59	NVG-7888	***Tiana niger***			Costa Rica: ACG, 1220 m, 10.92918, -85.46426, eclosed on 12-Aug-2011	USNM	11-SRNP-35371
60	NVG-7877	***Pellicia arina***			Costa Rica: ACG, 96 m, 10.96187, -85.28045, eclosed on 18-Mar-2013	USNM	13-SRNP-67590
61	NVG-7975	***Gorgopas trochilus***			Peru: Cuzco, Cosnipata Road, Pilcopata, 564 m, Stephen Kinyon, 15-Oct-2008	USNM	

Collection abbreviations

AMNH	American Museum of Natural History, New York, NY, USA
Brockmann	Research collection of Ernst Brockmann, Lich, Germany
LACM	Los Angeles County Museum of Natural History, Los Angeles, CA, USA
MNHP	Muséum National d'Histoire Naturelle, Paris, France
USNM	National Museum of Natural History, Smithsonian Institution, Washington, DC, USA
ZMHB	Museum für Naturkunde, Berlin, Germany
